# The surface condition effect of Cu_2_O flower/grass-like nanoarchitectures grown on Cu foil and Cu film

**DOI:** 10.1186/1556-276X-8-445

**Published:** 2013-10-28

**Authors:** Lijiao Hu, Yang Ju, Atsushi Hosoi, Yongpeng Tang

**Affiliations:** 1 Department of Mechanical Science and Engineering, Nagoya University, Furo-cho, Chikusa-ku, Nagoya 464-8603, Japan

**Keywords:** Flower/grass-like, Thermal oxidation, Nickel catalyst, Compressive stress

## Abstract

Cu_2_O flower/grass-like nanoarchitectures (FGLNAs) were fabricated directly on two category specimens of Cu foils and Cu film using thermal oxidation method. The FGLNAs are approximately 3.5 to 12 μm in size, and their petals are approximately 50 to 950 nm in width. The high compressive stress caused by a large oxide volume in the Cu_2_O layer on the specimen surface played an important role in the growth of FGLNAs. The effects of surface conditions, such as the surface stresses, grain size, and surface roughness of Cu foil and Cu film specimens, on the FGLNA growth were discussed in detail.

**PACS:**

81. Materials science; 81.07.-b Nanoscale materials and structures: fabrication and characterization; 81.16.Hc Catalytic methods

## Background

Cuprous oxide (Cu_2_O) is a p-type semiconductor metal oxide with a direct band gap of approximately 2.17 eV [1,2]. Due to its unique optical, electrical, and magnetic properties [3-5] and other properties such as simplicity and low cost of preparation, nontoxic nature, and abundance, it has attracted great attention and has been widely applied in solar energy conversion [6], photocatalysis [7], sensors [8], and antibacterials [9]. The fundamental properties of micro/nanostructure semiconductors are found to be dependent on their architectures, including geometry, morphology, and hierarchical structures [10-12]. Therefore, great efforts have been devoted to artificially control the morphology of Cu_2_O micro/nanocrystals in the past several years [13]. Different Cu_2_O nanoarchitectures have been synthesized, such as nanowhiskers [14], nanowires [11], nanocubes [15], nanorods [16], nanospheres [17], and nanoflowers [18]; Cu_2_O flower/grass-like three-dimensional nanoarchitectures (FGLNAs) with relatively large surface area have received particular attention and are expected to display significant semiconductor properties.

Various methods have been reported to synthesize Cu_2_O nanoflowers, such as pulse electrodeposition [19], polyol process [20], and solution-phase route [21]. However, up to now, all the fabrication methods of Cu_2_O flower-like architectures are complex and costly. Recently, we proposed a novel method using thermal oxidation with participation of catalyst and humidity to fabricate three-dimensional Cu_2_O FGLNAs (Hu LJ, Ju Y, Chen MJ, Hosoi A, and Arai S, unpublished observations). In the present paper, the growth mechanism of Cu_2_O FGLNAs affected by the surface conditions of different substrates was investigated in detail. The effect of surface stresses on the growth of FGLNAs - in unpolished Cu foil, polished Cu foil, and Cu film specimens before thermal oxidation - was analyzed. The effects of grain size and surface roughness of polished Cu foil specimens and Cu film specimens before heating were also studied.

## Methods

Two categories of specimens were prepared. One was made of a commercial Cu-113421 sheet (99.96% purity) with a thickness of 0.30 mm, which was cut into a square size of 6 × 6 mm^2^. Firstly, Cu foil specimens were put into diluted hydrochloric acid to get rid of the surface oxide on the specimens. Then, all the specimens were ultrasonically (Bransonic 1510, Branson Ultrasonics Corp., Danbury, CN, USA) cleaned and polished using abrasive paper. Five Cu foil specimens were polished using abrasive papers with 180, 240, 400, 800, and 1,000 grit, respectively. The other category specimens were coated Cu thin films on Cu foil through electrochemical deposition in the electrochemical cell containing 0.4 M copper sulfate pentahydrate and sulfuric acid (adjusting to desired pH 2) aqueous solution at a current speed of 15 mA/cm^2^ for 60 min. The temperature of the bath was maintained at room temperature. The surface state of the unpolished Cu foil, polished Cu foil, and Cu film specimens was measured by atomic force microscopy (AFM) and scanning electron microscopy (SEM, JSM-7000FK, JEOL Ltd., Akishima, Tokyo, Japan), and the surface roughness was also analyzed. Meanwhile, the surface stress of all the specimens was measured using the X-ray sin^2^ψ method by X-ray diffraction (XRD).

Afterwards, Ni catalyst was manually daubed on the surface of specimens as the shape of islands with a diameter of around 2 to 3 mm and thickness of 1 mm approximately. The nickel catalyst used in this experiment was a high-temperature resistance electrically conductive coating material (service temperature of 538°C, Pyro-DuctTM 598-C, Aremco, Inc., Valley Cottage, NY, USA). Specimens were then heated by a ceramic heater in air atmosphere under the humidity of 55% to 75% at the temperatures of 120°C and 240°C for 1, 2, and 3 h, respectively.

After the heating process, morphologies of FGLNAs grown on the specimens were characterized by SEM, energy-dispersive X-ray (EDX), and XRD.

## Results and discussion

**Figure 1:**
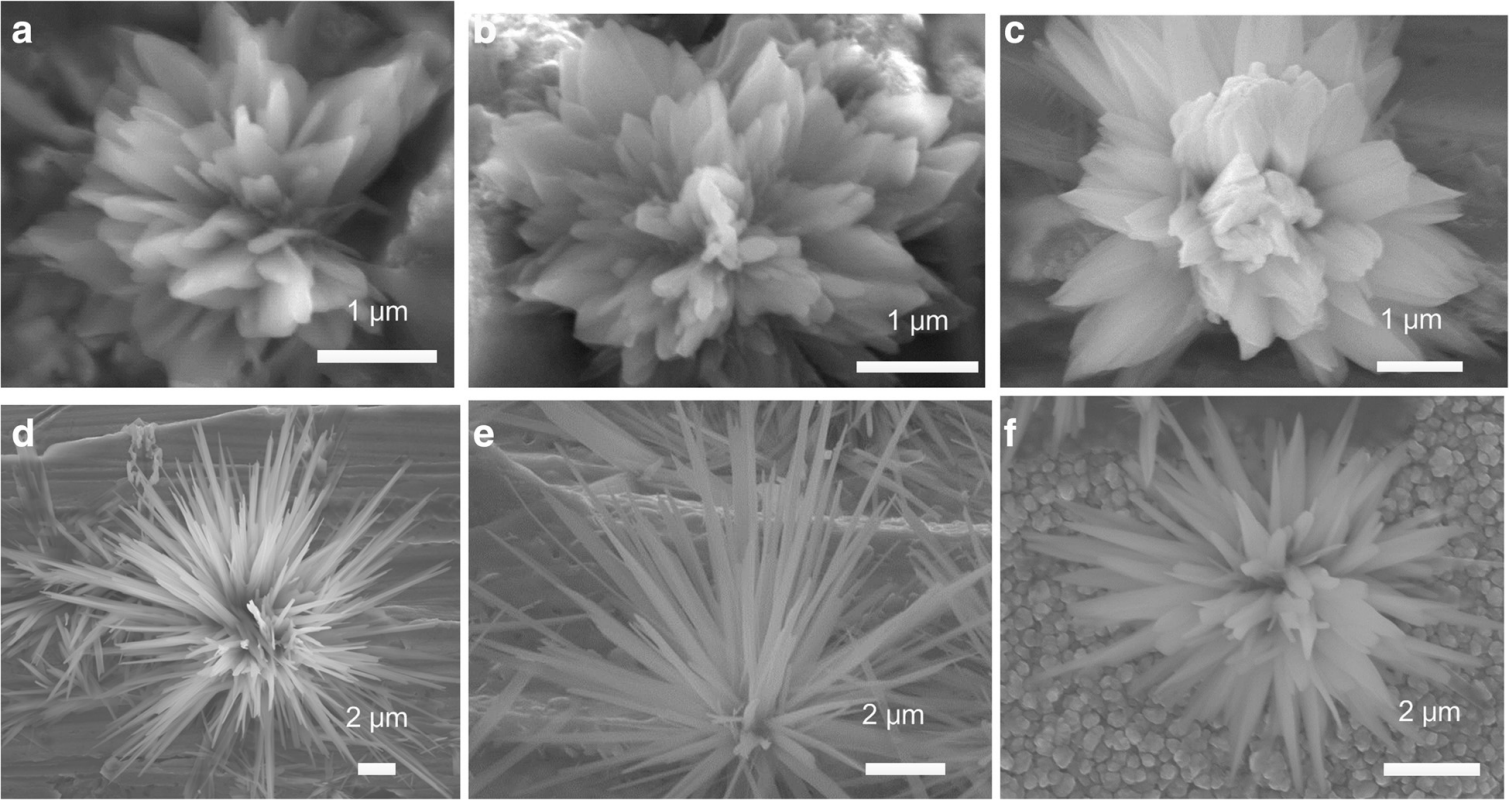
**SEM images of flower-like and grass-like architectures.** Flower-like architectures grown on **(a)** unpolished Cu foil specimen, **(b)** Cu foil specimen polished using a 400-grit abrasive paper, and **(c)** Cu film specimen heated at 120°C for 2 h, respectively. Grass-like architectures on **(d)** unpolished Cu foil specimen, **(e)** Cu foil specimen polished using a 400-grit abrasive paper, and **(f)** Cu film specimen heated at 240°C for 2 h, respectively.

As shown in Figure 1, the FGLNAs grow on the unpolished Cu foil, polished Cu foil, and Cu film substrates after heating at 120°C and 240°C for 2 h. The size of FGLNAs is 3.5 to 12 μm, and the width of their petals is 50 to 950 nm. A heating temperature of 120°C leads to generate flower-like architectures and 240°C leads to generate grass-like architectures. The different heating temperatures induce different stress migration and oxidation speeds, thereby leading to different structures of FGLNAs. It has been confirmed experimentally that there was no FGLNA growth when the experimental conditions were changed to vacuum environment, without catalyst or under the humidity lower than 55% or higher than 75%, respectively. Therefore, it is thought that besides temperature, oxygen atmosphere, catalyst, and humidity were three essential conditions for the growth of FGLNAs.

**Figure 2:**
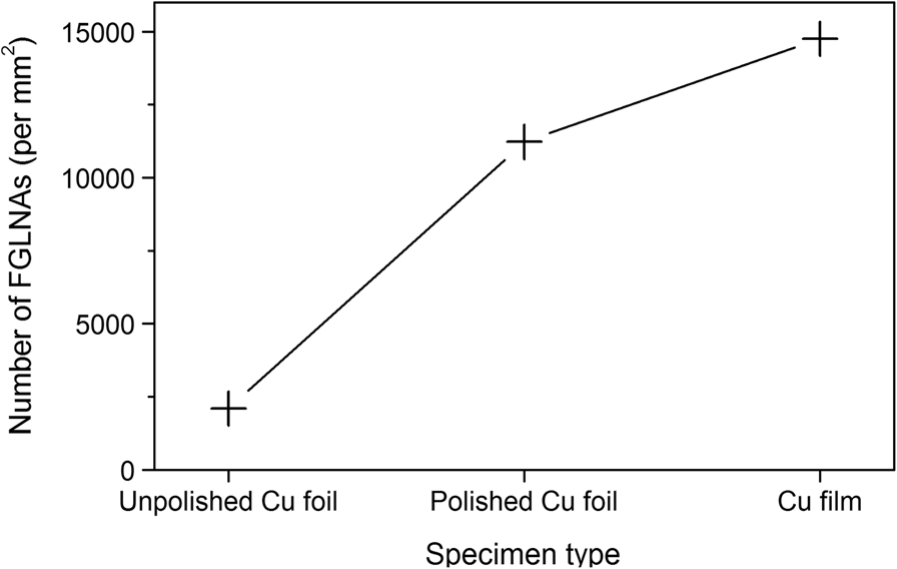
**Density of FGLNAs.** The FGLNAs were grown on unpolished Cu foil, polished Cu foil (400 grit), and Cu film specimens heated at 120°C for 2 h.

In addition, it was found that all the FGLNAs grown on different substrates have a similar shape and size for the same heating conditions. However, the density of FGLNAs is clearly different. The density of FGLNAs grown on unpolished Cu foil, Cu foil polished using a 400-grit abrasive paper, and Cu film specimens is shown in Figure 2. The densities of FGLNAs grown on the Cu film specimen and polished Cu foil specimen using a 400-grit abrasive paper are much higher than those grown on the unpolished Cu foil specimen. For all the polished foil specimens, the final results turned out that the best polishing condition for the growth of FGLNAs is 400 grit. The density of FGLNAs grown on the 400-grit polished Cu foil specimen is the highest among all the polished Cu foil specimens.

**Figure 3:**
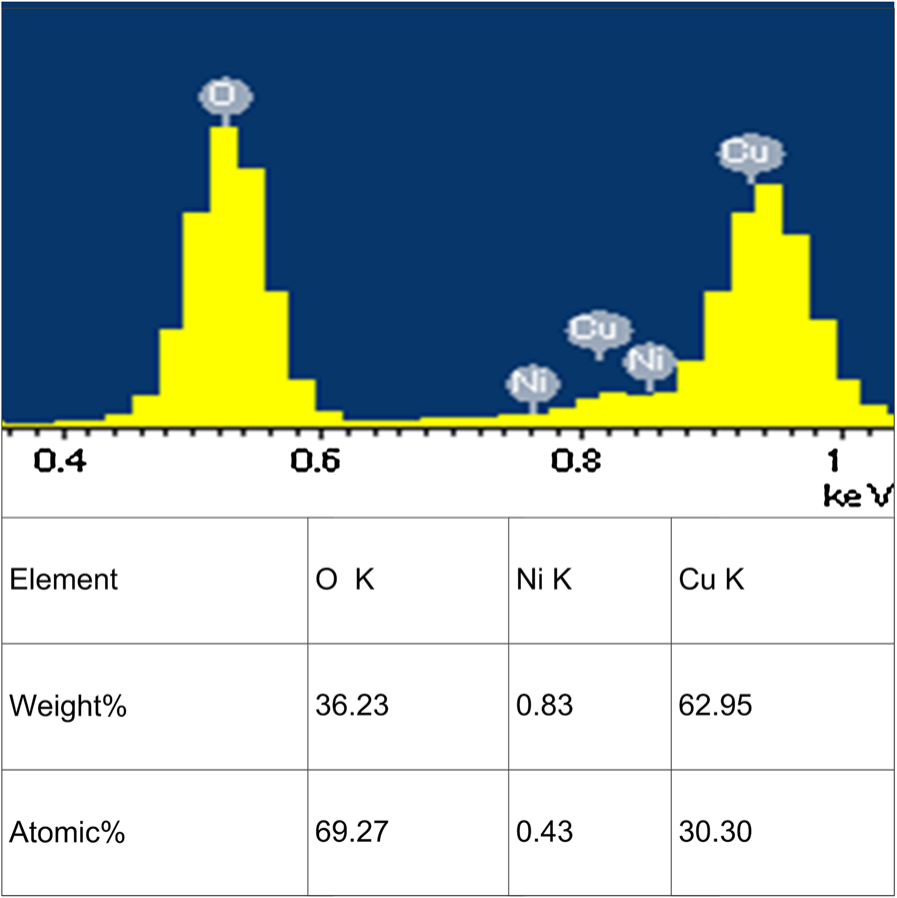
**EDX spectra of FGLNAs.** The FGLNAs were grown on the polished Cu foil specimen (400 grit) heated at 240°C for 2 h.

**Figure 4:**
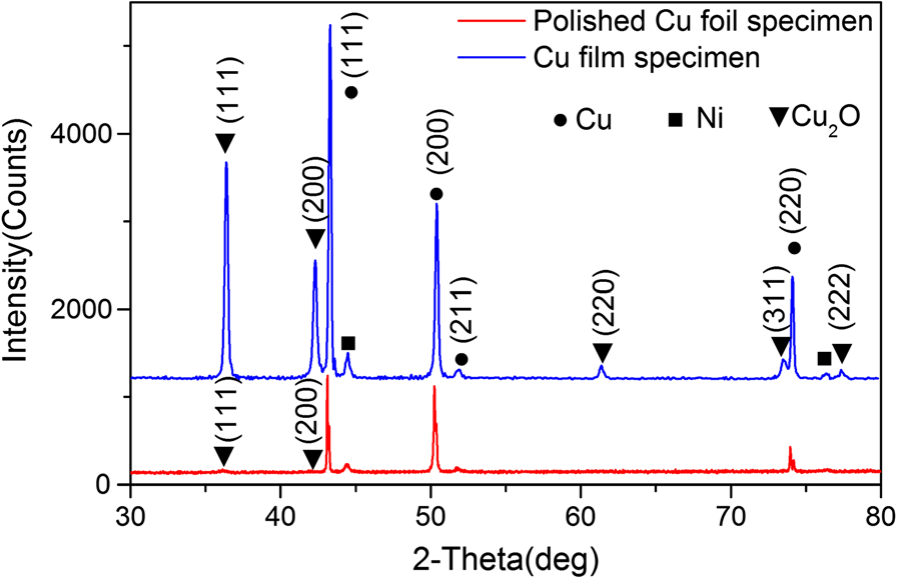
**XRD spectra of FGLNAs.** The FGLNAs were grown on polished Cu foil (400 grit) and Cu film specimens heated at 240°C for 2 h.

Figure 3 shows EDX analysis of the FGLNAs grown on the 400-grit polished Cu foil specimen heated at 240°C for 2 h. It indicates that the FGLNAs are mainly composed of the Cu element (30.30%) and oxygen element (69.27%). We also obtained similar EDX results for the other specimens. As shown in the XRD spectrum in Figure 4, orientations 111, 200, 311, etc. of Cu_2_O indicate that the FGLNAs are composed of Cu_2_O. Similar results of the XRD spectra were also obtained from the other specimens. As shown in the XRD spectrum, Ni is not oxidized. The reason is that the catalyst we used here is high-temperature resistance Ni; therefore, after heating, it continues to maintain as Ni.

**Figure 5:**
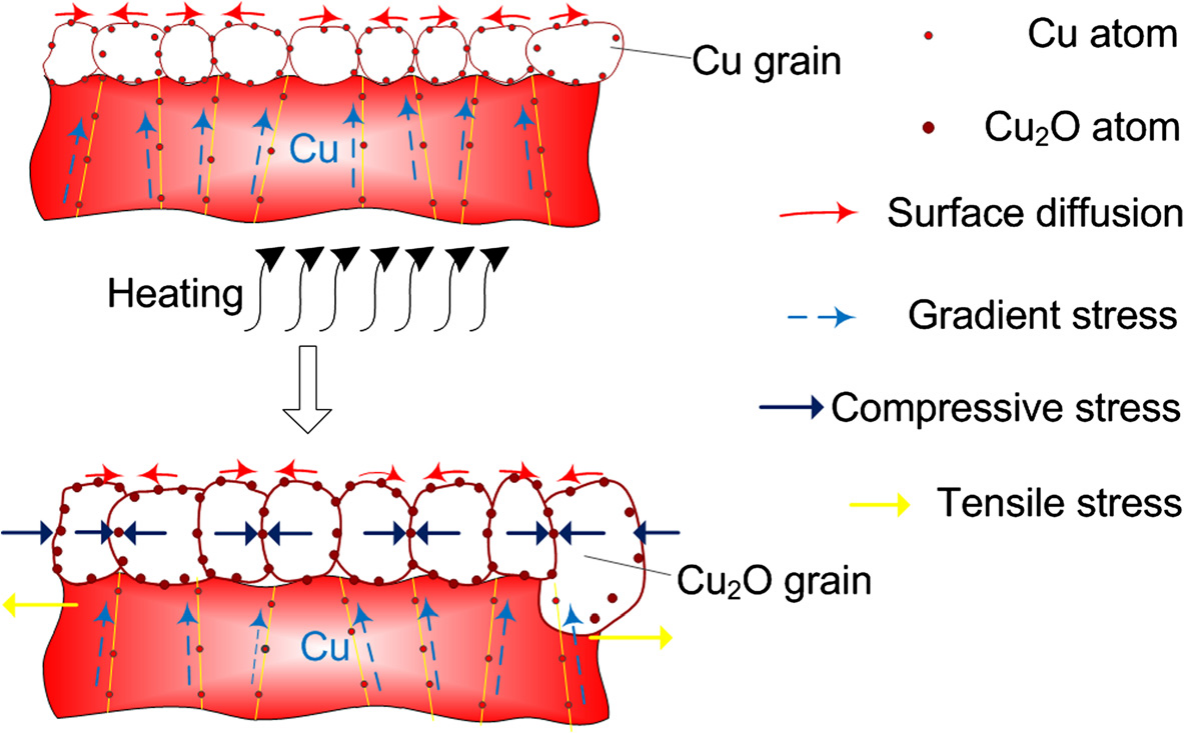
Illustration of stress generation mechanism due to the volume expansion of oxide layer.

When the specimens were heated in air, a Cu_2_O oxide layer formed on the surface of the specimens. As shown in Figure 5, compressive stress occurred in the oxide layer due to the oxide volume expansion. Meanwhile, as a reactive force, tensile stress occurred in the Cu substrate at the interface of Cu_2_O/Cu, which leads to the generation of vertical gradient stress (VGS) in the thickness direction of the specimen. Therefore, Cu atoms diffuse from the center of the Cu substrate to the interface between the oxide layer and the substrate due to the VGS. In the initial stage, since the temperature is relatively low (120°C and 240°C), the surface oxidation of the Cu foil/film is carried out under a low speed. The Cu_2_O layer that formed on the Cu foil/film is very thin, and the VGS is not large enough. Therefore, the diffused Cu atoms cannot penetrate the oxidation layer. However, with the participation of a catalyst and humidity, sufficient bivalent oxygen ions with two chemical bonds (BOICBs) were generated from the water vapors during the process of hydrogen absorption of the nickel catalyst, as indicated in Equation 1.

(1) $$H-O-H\overset{\Delta }{\underset{\mathrm{Ni}}{\to }}-O-+H_{2}$$

Thus, the low-temperature oxidation was enhanced, and the thickness of the Cu_2_O layer became larger and larger. Therefore, the compressive stress in the Cu_2_O layer caused by oxide volume expansion will be larger than the results without participation of catalyst and humidity, thereby creating larger VGS. On the other hand, the compressive stress in the oxide layer also made it difficult for Cu atoms to penetrate through the oxide layer from the weak spots on the surface. Consequently, Cu atoms kept accumulating under the oxide layer until there were enough Cu atoms to break the balance, and finally, a large number of Cu atoms suddenly penetrated the oxide layer through the weak spots in a flash. It is noted that since the surface Cu_2_O layer was relatively thicker, which leads to a small number of weak spots and requires a relatively large penetration force, a large number of Cu atoms accumulated and penetrated the Cu_2_O layer through the same weak spots. Cu atoms burst out and are more easily oxidized. The formation of a nanostructure is to make Cu atoms perfectly disperse into a 3-D space, which are typically manifested as flower and grass architectures in nature. Moreover, the BOICBs served as a nuclear site during the formation of FGLNAs. Firstly, BOICBs bound Cu atoms together. Then, Cu atom oxide and Cu_2_O atoms realign and grow into the shape of petals/leafage. Finally, petals/leafage incorporates and forms into FGLNAs. Therefore, VGS and BOICBs are two key factors for the growth of FGLNAs. It should also be noted that the mechanism of VGS created in the Cu foil/film here is different from that in the Cu film on the Si substrate [10,22,23] in which the VGS generated due to the thermal expansion mismatch of the materials. That is the reason that Cu_2_O FGLNA growth under a relatively low temperature was realized, instead of CuO nanowire growth under a relatively high temperature.

**Figure 6:**
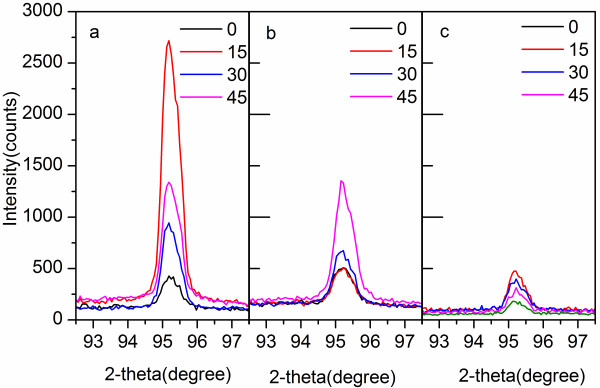
**Ex situ *θ*/2*θ *diffractograms measured for X-ray stress analysis. (a)** Unpolished Cu foil, **(b)** polished Cu foil (400 grit), and **(c)** Cu film specimens before heating. The legend reports the corresponding ψ angles (i.e., inclination of the specimen).

**Figure 7:**
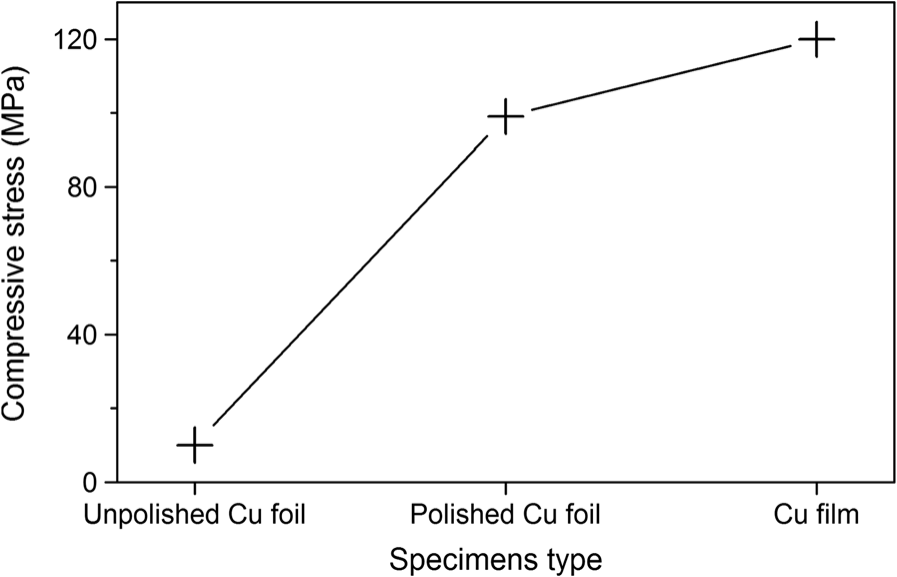
X-ray stress of unpolished Cu foil, polished Cu foil (400 grit), and Cu film specimens before heating.

**Figure 8:**
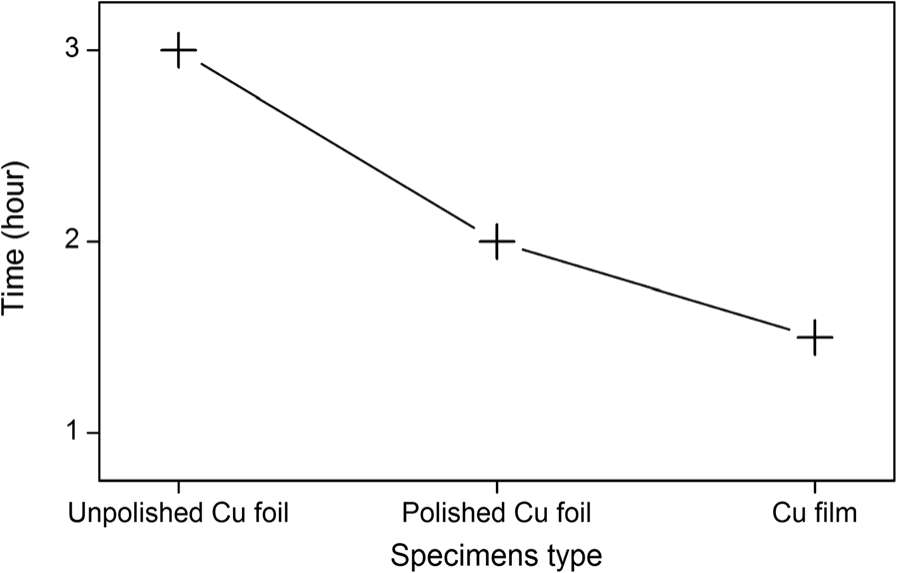
**Heating time for the first appearance of FGLNAs.** The FGLNAs were grown on the specimens of unpolished, polished Cu foils (400 grit), and Cu film.

To further investigate the effect of surface conditions on the generation of FGLNAs, the X-ray sin2ψ method [24] was used to measure the residual stresses in unpolished Cu foil, polished Cu foil (400 grit), and Cu film specimens before thermal oxidation, respectively. Before heating, the X-ray diffraction (sin2ψ) method was employed using the {222} diffraction Cu peak, occurring at a diffraction angle of approximately 2*θ* = 95.2°. As shown in Figure 6, slow step scanning in the range of approximately 92.5° to 97.5° of 2*θ* was conducted for ψ-angles in the range of 0° to 45°. Based on the results of Figure 6, the stresses were calculated using JADE software (version 6.5). As shown in Figure 7, compressive stresses were measured for unpolished Cu foil, polished Cu foil (400 grit), and Cu film specimens to be 10, 99, and 120 MPa, respectively. Therefore, the polishing put a pre-stress in the Cu foil specimen. During polishing, the grits of abrasive paper squeeze the surface of the Cu foil and rub it into the rough surface which will leave a compressive residual stress on the surface of the polished Cu foil specimen [25]. It can be found that Figure 7 has a similar shape with Figure 2, which indicates that the initial compressive stress on the specimen surface has a relationship with the density of FGLNAs grown on the specimen. It is considered that initial compressive stress has an action to obstruct the volume expansion of the oxide layer which formed on the specimen surface during the heating process. Therefore, a higher effective VGS would occur for the same oxide volume expansion, which induces more and faster diffusion of Cu atoms to the specimen’s surface, thereby increasing the density of grown FGLNAs. On the other hand, the heating time for the first appearance of FGLNAs was also observed for the specimens of unpolished Cu foil, polished Cu foil (400 grit), and Cu film. As shown in Figure 8, the heating time for the specimens of unpolished Cu foil, polished Cu foil (400 grit), and Cu film is 3, 2, and 1.5 h, respectively. Compared with the results shown in Figure 7, higher initial compressive stress in the specimen leads to shorter heating time for the first appearance of FGLNAs. It indicates that higher vertical gradient stress promotes the diffusion of Cu atoms, thereby speeding up the growth of FGLNAs. Therefore, the same heating time results in the highest density of FGLNAs grown on the Cu film specimen. Moreover, the thickness of the Ni catalyst can also affect the growth time of Cu_2_O FGLNAs but does not affect the morphology and size. Thinner thickness of the Ni film would lead to a longer time for the growth of FGLNAs.

**Figure 9:**
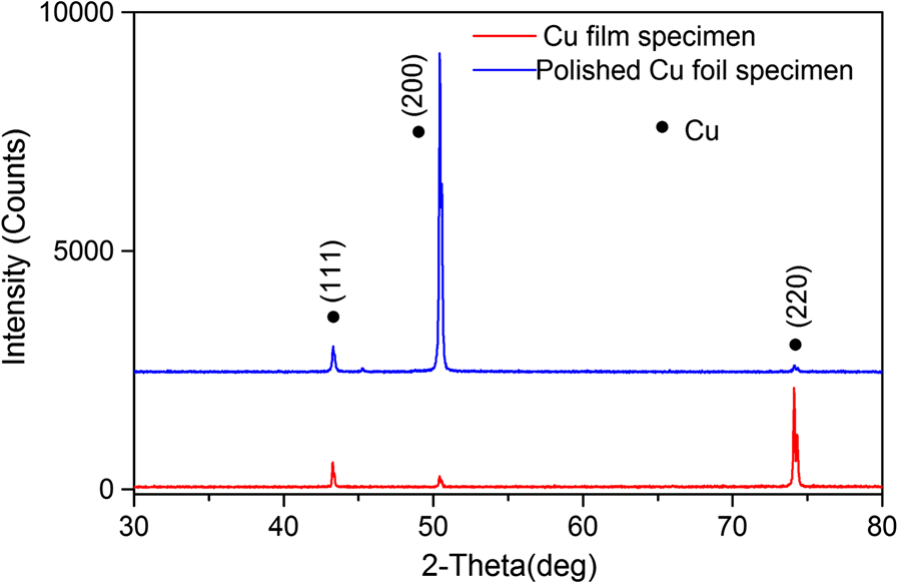
XRD spectra of polished Cu foil (400 grit) and Cu film specimens before heating.

Figure 9 shows the XRD spectra of polished Cu foil (400 grit) and Cu film specimens before heating, and the peak width at half height was calculated using the JADE software (version 6.5). Mean grain size determined from the width of the diffraction peaks using Scherrer’s formula is 42 nm for the specimen of polished Cu foil and 59 nm for the Cu film specimen. It is considered that larger grain size may induce larger initial compressive stress in the specimen, thereby creating larger vertical gradient stress to promote the growth of FGLNAs. It should be noted that polishing would not change the crystal size of the Cu foil specimen. Therefore, the crystal size of the polished Cu foil specimen is the same as that of the unpolished Cu foil specimen.

**Figure 10:**
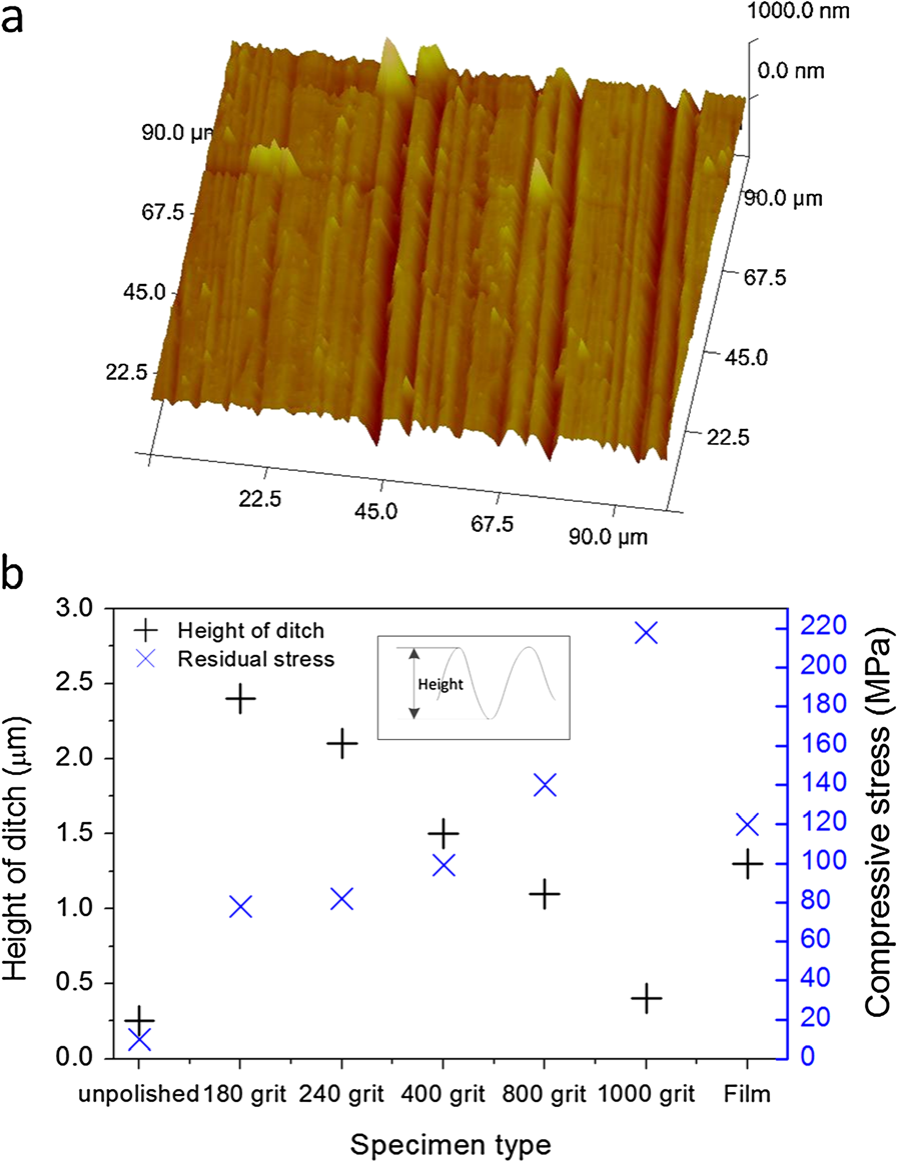
**AFM topography image, surface ditch height, and residual stress. (a)** AFM three-dimensional topography image of the unpolished Cu foil specimen. **(b)** Surface ditch height and residual stress of unpolished Cu foil, polished Cu foil, and Cu film specimens.

In addition, surface roughness is believed to have an effect on the growth of FGLNAs. Surface topography of unpolished Cu foil, polished Cu foil, and Cu film specimens was measured by AFM, and the surface roughness was evaluated using the height of ditches, as shown in Figure 10. To compare with the stress condition, measured initial residual stress on the specimen surface before heating is also shown in Figure 10. It can be found that the 400-grit polishing specimen has a similar roughness as the Cu film specimen (around 1.4 μm). It was suspected that the surface roughness may increase the surface area, thereby promoting the surface oxidation of the specimen (i.e., enhancing VGS), and there is an optimum value for the growth of FGLNAs. It also can be found that the measured compressive stresses for the specimens of 800 and 1,000 grits polished are greatly larger than that of the 400-grit polished specimen. The reason why high-density FGLNAs were not observed on these high initial stress specimens is that the relatively low surface roughness may lack enough surface area to further enhance the growth of FGLNAs on the specimens. Therefore, there is a balance between the initial compressive stress and surface roughness for the growth of FGLNAs.

## Conclusions

Cu_2_O FGLNAs which are 3.5 to 12 μm in size with 50- to 950-nm wide petals were successfully fabricated using the thermal oxidation approach with catalyst under moderate humid atmosphere. The effect of surface conditions, such as surface stress, grain size, and roughness, on the growth of FGLNAs was analyzed. Larger initial compressive stress, optimum grain size, and surface roughness were beneficial for the formation of FGLNAs. Compared with other methods for fabricating Cu_2_O FGLNAs, the thermal oxidation method featured remarkable simplicity and cheapness.

## Abbreviations

FGLNAs: flower/grass-like nanoarchitectures; BOICBs: bivalent oxygen ions with two chemical bonds; VGS: vertical gradient stress.

## Competing interests

The authors declare that they have no competing interests.

## Authors’ contributions

LJH designed and performed all the experiments, analyzed the data, and wrote the main manuscript text. YJ designed and conducted the whole study. AH and YPT performed the AFM characterization experiments. All authors reviewed the manuscript. All authors read and approved the final manuscript.
